# Traditional Chinese medicine fumigation as auxiliary treatment of diabetic peripheral neuropathy: A protocol for systematic review and meta-analysis

**DOI:** 10.1097/MD.0000000000024200

**Published:** 2021-01-29

**Authors:** Shixin Kang, Yanmei Zhong, Donghao Liu, Weihong Li

**Affiliations:** a School of Basic Medical Sciences, Chengdu University of Traditional Chinese Medicine; b School of Medical Information Engineering, Chengdu University of Traditional Chinese Medicine, Chengdu, Sichuan Province, China.

**Keywords:** diabetic peripheral neuropathy, effectiveness and safety, protocol, systematic review, traditional Chinese medicine fumigation

## Abstract

**Background:**

Diabetic peripheral neuropathy (DPN) is 1 of the most common clinical complications of diabetes, which seriously affects the quality of life of patients and causes a substantial economic burden on diabetes care. The pathogenesis of DPN is complex. There is no targeted treatment method, and mainstream treatment methods have low efficacy and large side effects. Traditional Chinese medicine has rich clinical experience in the prevention and treatment of diabetic peripheral neuropathy, which has dramatically improved the quality of life of patients. It is clinically proven that traditional Chinese medicine fumigants (TCMF) have apparent effects in treating diabetic peripheral neuropathy. Therefore, we aim to systematically review the effectiveness and safety of TCMF for DPN.

**Methods:**

We will search the following databases: PubMed, Embase, Cochrane Library, MEDLINE, the China National Knowledge Infrastructure, the Chinese Biomedical Literature Database, Cqvip Database, and Wanfang Data. Besides, we will also search for clinical trial registrations, potential grey literature, relevant conference abstracts, and reference lists of established studies. The studies published from the inception of the database to November 2020 will be retrieved. The randomized controlled trials on TCMF for DPN will be included. Also, we will search for clinical trial registrations, potential grey literature, relevant conference abstracts, and reference lists of established studies. The main result is clinical efficacy and nerve conduction velocity. Fasting blood glucose, 2 hours postprandial blood glucose, blood lipid, glycosylated hemoglobin, and adverse events are secondary results. We will perform the analyses using RevMan V.5.3 software.

**Results:**

This study will provide a high-quality comprehensive evaluation of the efficacy and safety of TCMF in the treatment of DPN.

**Conclusions:**

This systematic review will evaluate the effectiveness and safety of TCMF in the treatment of DPN, and provide the latest evidence for clinical application.

**INPLASY registration number:**

INPLASY2020110137.

## 1 Introduction

Diabetes is currently 1 of the most severe chronic diseases in the world, threatening the lives and health of millions of people.^[1]^ At the same time, diabetic peripheral neuropathy (DPN) is 1 of the most common complications of diabetes, and about half of diabetic patients will be affected.^[2]^ “The presence of symptoms and/or signs of peripheral nerve dysfunction in people with diabetes after the exclusion of other causes.”^[3]^ This is a simple clinical definition of DPN. It mainly affects sensory nerve and motor function. Clinical manifestations include: neuropathic pain, paresthesia, hyperesthesia or sensory decline.^[4]^ There are many ways to detect DPN,^[5]^ but nerve conduction studies are usually considered as the diagnostic standard.^[6]^ It is clinically affected by a variety of risk factors, and its pathogenesis is complex.^[7,8]^ Furthermore, as the course of the disease progresses, DPN can lead to foot infections and even severe consequences of amputation.^[9]^ This is usually a heavy burden for the healthcare system and patients.^[10]^

At present, the primary treatment for DPN is still drug treatment, mainly symptomatic treatment, such as blood sugar control, but still can’t achieve satisfactory results.^[11,12]^ Other drugs such as various painkillers have been evaluated in DPN, but due to the various side effects of the drugs, patient compliance is often low.^[13,14]^ Therefore, finding the best treatment for DPN is a challenging task for doctors.^[13]^

In recent years, Traditional Chinese medicine has played an important role in the prevention and treatment of severe chronic diseases in the world. Clinically, a unique treatment method is used to treat diabetic peripheral neuropathy, and the traditional Chinese medicine fumigation treatment is 1 of the representatives. TCMF can directly act on the diseased part of the patient, accelerate the blood circulation of the diseased part, and promote the improvement of the nerve function of the foot and the clinical symptoms. It is now widely used in the treatment of DPN and has achieved good results.^[15–17]^

## 2 Methods

### 2.1 Study registration

The protocol for this systematic review was registered on INPLASY (INPLASY2020110137) and is available in full on the inplasy.com (https://doi.org/10.37766/inplasy00000000).

### 2.2 Ethics and dissemination

All data for this systematic review protocol have been published online, and therefore the ethical approval is not needed.

### 2.3 Inclusion criteria

#### 2.3.1 Types of studies

All randomized controlled trials (RCTs) about TCMF for DPN will be included regardless of language. The following studies: case series, quasi-RCTs Case reports, non-RCTs, cell experiments, animal experiments will be excluded.

#### 2.3.2 Participants

DPN patients must meet the diagnostic criteria of the “China Type 2 Diabetes Prevention Guidelines” issued by the Diabetes Branch of the Chinese Medical Association in 2017,^[18]^ regardless of race, gender and age. Neuropathy caused by other reasons and severe chronic wasting disease, pregnant and lactating patients is not included.

#### 2.3.3 Types of interventions

Both groups used the conventional diabetes treatment methods recommended by the ADA guidelines, including diet, exercise, hypoglycemic and lipid-lowering therapy.^[19]^ The experimental group was treated with TCMF, while the control group applied for placebo, neurotrophic drugs, or no treatment. In addition, the 2 groups did not take any drugs that would affect the outcome indicators. Follow-up time ≥ 4 weeks.

#### 2.3.4 Types of outcome measures

The improvement in clinical efficacy and nerve conduction velocity is the main result. The clinical efficacy refers to the guiding principles for clinical research of new Chinese medicines,^[20]^ the specific evaluation criteria are as follows: markedly effective: symptom improvement> 70%; effective: symptom reduction 30% to 70%; ineffective: symptom improvement <30%, or no improvement, even worse. Nerve conduction velocity is evaluated using electromyography.

The secondary results mainly consist of fasting blood glucose, 2 hours postprandial blood glucose, blood lipids, glycosylated hemoglobin and adverse events.

### 2.4 Exclusion criteria

We will exclude the following documents, including: studies where complete data are not available; studies with data errors; studies using wrong intervention methods or random methods. For duplicate documents, we will choose 1 of them.

### 2.5 Search strategy and study selection

#### 2.5.1 Search strategy

The following electronic databases will be comprehensively searched including: PubMed, Cochrane Library, EMBASE, MEDLINE, China National Knowledge Infrastructure, Biomedical Literature Database, Cqvip Database, Wanfang Data, World Health Organization International Clinical Trials Registry Platform, Chinese Clinical Trial Register, Clinical Trials, and Grey Literature Database. all the literature retrieved is from the time when the database establishment to 20 November 2020. There are no language restrictions or regional restrictions. The following search terms were used individually or in combination: “diabetic peripheral neuropathy,” “Traditional Chinese medicine fumigation,” and “randomized controlled trial.” We will simply present the search process of PubMed (Table 1). Adjusting different search methods according to different Chinese and English databases.

**Table 1:** Search Strategy for PubMed.

1	diabetic neuropathy [MH]
2	diabetic peripheral neuropathy [ALL]
3	diabetic neuropathies [ALL]
4	DPN [ALL]
5	1 OR 2 OR 3 OR 4
6	traditional Chinese medicine [MH]
7	Drugs, Chinese Herbal [MH]
8	traditional Chinese herbal medicine [ALL]
9	Chinese herb∗[ALL]
10	6 OR 7 OR 8 OR 9
11	Fumigation [MH]
12	Fumigations [TIAB]
13	11 OR 12
14	10 AND 13
15	randomized controlled trial[PT]
16	controlled clinical trial[PT]
17	randomized [TIAB]
18	placebo [TIAB]
19	randomly [TIAB]
20	15 OR 16 OR 17 OR 18 OR 19
21	5 AND 14 AND 20

#### 2.5.2 Study selection

**Figure 1:**
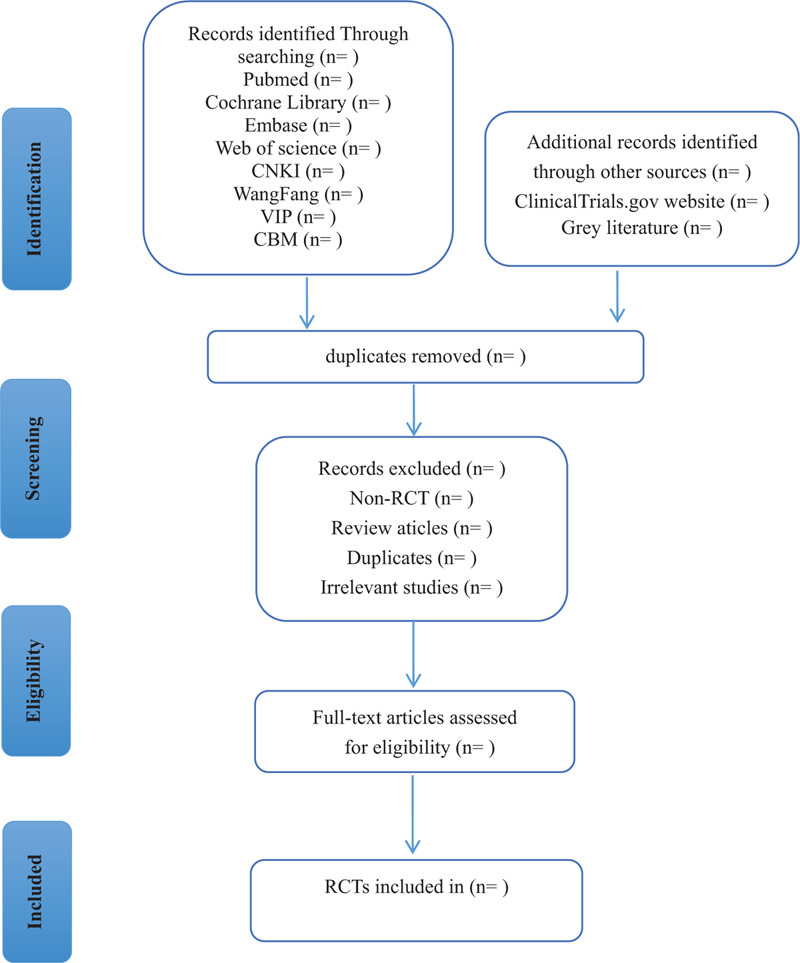
Flow diagram of the study selection process.

Two researchers will independently search all relevant documents and import them into EndNote X9 software for management. Two independent researchers first read the title and abstract to eliminate duplicate or irrelevant documents, and finally read the full text to confirm eligible studies. If any dispute occurs, the 2 researchers will discuss and reach an agreement. If no consensus is reached, the disagreement will be resolved by consulting a third researcher. Missing literature information will be supplemented by contacting the original author. The research selection process will strictly follow the PRISMA flowchart. (Fig. 1).^[21]^

### 2.6 Data extraction

Two researchers independently completed the evaluation of the research according to the conformity assessment form and extracted the research results. The extracted data includes author, gender, age, publication date, country/region, sample size, intervention details, follow-up information, safety, results, and so on. The above information will be repeatedly checked by 2 researchers, and any disputes about data extraction will be resolved through negotiation.

### 2.7 Assessment of risk of bias

The Cochrane collaborative tools will be used to assess the risk of literature bias.^[22]^ Two investigators used RevMan 5.3.0 to assess method quality independently. Evaluate the following 7 aspects, including: randomness, the blindness of participants and researchers, sequence generation, allocation hiding, blindness of result evaluation, selective result reporting, incomplete result data and other biases. The quality of each experiment was assessed as low, unclear, or high biased. Resolve differences through discussion between the 2 reviewers or seeking third-party consultation.

### 2.8 Statistical analysis

For data analysis, RevMan 5.3.0 that is provided by the Cochrane Collaboration will be used. We will use the chi-square test and *I*^2^ statistic to evaluate the heterogeneity of similar studies. If *P* ≥.05 and *I*^2^≤ 50%, we believe it is low heterogeneity. As a result, we will use a fixed-effects model. If *P*<.05 and *I*^2^ > 50%, it means there is heterogeneity. we will use a random-effects model. For the enumeration data, odds ratio (OR) with a 95% confidence interval will be used to represent. We will use mean difference with 95% confidence interval to express the measurement data. The statistically significant difference is thought of as *P* < .05.

If the studies show significant heterogeneity, we will conduct a subgroup analysis of different traditional Chinese medicine fumigation prescriptions to explore whether the Chinese medicine fumigation prescriptions cause heterogeneity. Furthermore, if necessary, a sensitivity analysis will be performed.

### 2.9 Publication bias

If more than 10 studies are included in the meta-analysis, we use a funnel chart to measure publication bias and carefully interpret the results.

## 3 Discussion

DPN is threatening the health of nearly half of diabetic patients.^[2]^ However, there is no safe and effective clinical treatment for DPN.^[11–13]^ What is more serious is that DPN can cause serious consequences and severe burden.^[9,10,23]^

Traditional Chinese medicine has rich experience in the treatment of diabetes and its complications, as well as various treatment methods. Clinical practice shows that TCMF can improve the symptoms of DPN and increase nerve conduction speed.^[15–17]^

However, there is no evidence-based medicine to prove the efficacy of TCMF on DPN. Therefore, we conduct a meta-analysis to provide a high-quality basis for the clinical effectiveness and safety of TCMF and hope to promote the development and application of Traditional Chinese medicine to benefit patients.

## Author contributions

**Conceptualization**: Shixin Kang, Weihong Li.

**Data curation**: Donghao Liu.

**Funding acquisition**: Weihong Li.

**Investigation**: Shixin Kang, Yanmei Zhong.

**Methodology**: Shixin Kang, Yanmei Zhong.

**Project administration**: Shixin Kang, Weihong Li.

**Software**: Donghao Liu.

**Supervision**: Yanmei Zhong, Donghao Liu.

**Validation**: Weihong Li.

**Writing – original draft**: Shixin Kang, Yanmei Zhong.

**Writing – review & editing**: Shixin Kang, Yanmei Zhong.

## Abbreviations

Abbreviations: DPN = diabetic peripheral neuropathy, RCTs = randomized controlled trials, TCMF =Traditional Chinese medicine fumigation.
